# Ohio Dental College Laboratory

**Published:** 1874-12

**Authors:** 


### OHIO DENTAL COLLEGE LABORATORY.

In order to give answer to some enquiries in reference to the course of instruction in this Institution, we copy from the memorandum book of the Professor of Mechanical Dentistry, the following particulars which will give some idea of the course in this department at least:

"Each student must provide for his own use all necessary tools and appliances. Also Lead, Zinc, Brass, Wax, and Silver solder. Every student is required to become thoroughly familiar with the following particulars and processes:

1st. Working wax, and plaster Paris.

2d. Taking impressions in wax from plaster models, filling them, removing from the wax impression, and preparing for moulding in sand.

3d. Moulding various models in sand with appropriate flasks.

4th. Preparing metals and casting models.

5th. Making contour models by various methods.

6th. Forming patterns for plates.

7th. Preparation of material for plate.

8th. Cutting out, modeling and swaging plates.

9th. Making plates with swaged chambers—Cleveland or cut chamber—Smith's method.

10th. Forming plate with turned and swaged rim.

11th. Making plate with stamped or burnished socket rim, also with band.

12th. Making articulating models of various forms.

13th. Selecting grinding and adjusting teeth.

14th. Investing dentures for soldering.

15th. Putting the linings or backings upon the teeth.

16th. Making silver solder.

17th. Management and use of the blow-pipe.

18th. Soldering teeth to the plate.

19th. Finishing.

20th. The various methods of securing partial dentures—clasps—atmospheric pressure.

21st. Adjusting and attaching teeth to partial plates.

22d. Working gold, refining, alloying—separating from other metals—hammering, rolling and drawing.

23d. Making gold solders,

24th. Continuous gum work.

25th. Adjusting and arranging teeth for same.

26th. Lining and soldering the teeth to the plate.

27th. Putting on body and biscuiting, putting on gum enamel and baking. Management of furnace.

28th. Taking impressions in the mouth with wax; with plaster —for full dentures—for partial dentures.

29th. Principles involved in articulating full single dentures to natural teeth; also partial dentures to natural teeth; also full double dentures.

30th. Method of modeling for restoring the features—attachment of appliances for restoration.

31st. Method of constructing vulcanite dentures.

32d. Method of making celluloid dentures.

33d. Method of making cast metal plate.

34th. Block work.

35th. Working and tempering steel.

